# A descriptive retrospective study of time consumption in home care services: how do employees use their working time?

**DOI:** 10.1186/1472-6963-14-439

**Published:** 2014-09-26

**Authors:** Solrun G Holm, Ragnhild O Angelsen

**Affiliations:** The Faculty of Professional Studies, University of Nordland, 8049 Bodø, Norway; Geodata AS, Schweigaardsgate 28, 0133 Oslo, Norway

**Keywords:** Home care services, Home care recipient, Geographic information systems (GIS), Nursing, Driving route, Driving time, Visiting time, Working time

## Abstract

**Background:**

Home care services in Norway are provided for free, and municipalities are responsible for their provision to all those in need of them, in accordance with the Act on Municipal Health and Care Services. The costs of home care services are increasing. Many municipalities are now working to find the best cost-effective solutions to ensure that home care services are of sufficient quality but still affordable. This paper describes how nurses and health workers spend their working time, with a hypothesis that driving time and time required to document details of the care given are underestimated in weekly planning schedules.

**Methods:**

This article sets out a descriptive retrospective study of day-schedules and driving routes for staff working in home care services. Data were analyzed using GIS.

**Results:**

The driving time was between 18% and 26% of working time in municipality A, and between 21% and 23% in municipality B. Visiting time varied between 44% and 62% in municipality A, and 40% and 56% in municipality B. Other tasks, including the legally-required documentation of the care given, varied between 19% and 32% in municipality A and 21% and 38% in municipality B. Overall, 22% of the driving routes in municipality A, and 14% in municipality B, took more time than expected. In municipality A, 22% of the day-schedules underestimated overtime; this figure was 14% in municipality B.

**Conclusions:**

In home care services, time taken for driving and to write statutory documentation seems to have been underestimated. Better planning and organization of driving routes would reduce driving time and allow more time for other necessary work.

## Background

The population in Norway is aging, more people need help for longer periods and more diseases are treatable with new technology [1]. Norway provides free health care and ranks among the highest of all OECD nations for public health spending per capita [2]. The Government believed that these developments were not sustainable, and, on this basis, introduced a new health care reform in 2012 called *The Coordination Reform: Proper treatment – at the right place and right time* [3]. To follow this up, a new law and regulations on health and care services were also introduced: *The Norwegian Health Service Act* [4]. *The Coordination Reform* and the new law changed municipalities’ role in the provision of coordinated health and care, giving them the largest part in meeting the growth in demand for health services. They are responsible for providing reasonable, high-quality home care services, including both social and nursing care, to all who need them, regardless of age or diagnosis. Developments in recent years show that home care recipients with comprehensive assistance needs have been assigned a growing number of hours of assistance per week [5]. One year after *The Coordination Reform* was implemented, the cost of home care services is increasing. Many municipalities are now working to find the most cost-effective solutions that will ensure that home care is of adequate quality but still affordable.

People who need home care services may apply for them to the municipality. All home care recipients are registered in an electronic patient journal system, which also records decisions about what services the municipality has assigned to each of them, including home help, home nursing care or both. The decisions show how many hours of home help or home nursing care each user should receive each week, and which tasks should be done in that time.

If the decision means that the recipient receives more than one visit per week, the time allocated is divided by the number of visits and the services to be performed. Employees in home care offices operationalize the decision, often in collaboration with home care recipients, to decide which days and at which time the visits will occur. Home care recipients often live far from home care services’ offices, which mean that nurses and health care workers need to use cars to visit them.

There is no limit to the number of home care recipients in any given municipality. The Act on Municipal Health and Care Services says only that the municipality must ensure that all persons residing there receive the necessary health services [4].

Lack of time is perceived as a problem for the staff in home care services. During the last few years there have been a lot of press reports about home care services, focusing on the lack of time, including “Stopwatch care” [6–8], “Tough priorities in home care services” [9], and “6 minutes for dinner help” [10]. It is also reported as an issue in several studies on homecare services. These studies have showed that nurses and health workers in Norway experienced increased time pressure and lack of time as a dominant and restricting factor in providing home care services [11–16].

Since home care recipients get a certain number of hours of home care services per week, providers set up day schedules and driving routes on a weekly basis. Traditionally, operational planning is done manually. A nurse or experienced health worker has the responsibility for organizing the weekly plan of day schedules and driving routes according to the sector’s record of recipients. This is quite a complex task, requiring those responsible to take into account the tasks to be done at each port of call, and the expertise required. Some visits might require more than one employee to provide comprehensive nursing. In addition, they must bear in mind that some tasks, such as the administration of medication like insulin, should happen at a fixed time. The day schedules and driving routes show how each sector operationalizes the care required for each home care recipient, and the amount of time employees are expected to spend with them. The list of driving routes does not show the driving time, only the visiting time.

Since research shows that nurses and other health workers in home care services experienced lack of time as a problem, this indicates that the planning of day schedules and driving routes is probably not optimal. Planners know the visiting time but not the driving time and transfer time from the car into the recipients’ house and back again.

Many articles discuss how to solve the planning problems for home health care services, and balance the workload and optimal driving routes [17–24]. Eveborn et al. [25] developed a system for home care services that included travel times and distances.

There is, however, no research that shows how much driving time employees of home care services are expected to use when they visit home care recipients, and only a few reports based on the employee's own record of how they spent time during the shift [26]. Using the geographic information system, GIS, we were able to register driving routes in home care in a GIS application for calculating driving time [20, 24].

There have been a number of studies using GIS in health care research. Nykiforuk and Flaman [27] carried out a literature review to identify how GIS applications have been used in health-related research. They found that in general, researchers had focused on the distance or travel time to health facilities [28–32] rather than time taken for home care services employees to travel to home care recipients.

The aim of our study is to learn how nurses and health workers in home care services spent their working time using GIS to analyze a weekly plan of daily schedules and driving routes, and in particular to calculate distance driven and the amount of time spent driving, in addition to visiting time. Our hypothesis is that driving time and time required to document details of the care given are underestimated in planning schedules.

## Methods

This is a descriptive, retrospective study of day-schedules and driving routes for employees of home care services in two municipalities in northern Norway.

**Figure 1:**
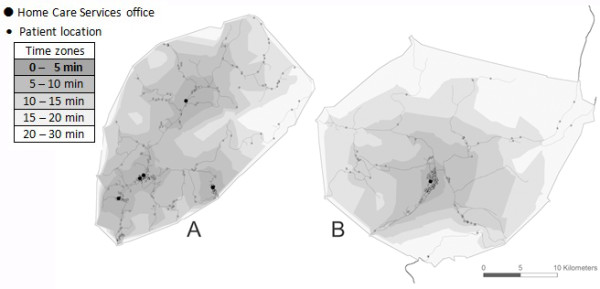
**Service area analysis: 5-, 10-, 15-, 20- and 30-minute travel time zones.** For office 5 in municipality **A** and office 4 in municipality **B**, the home care recipients all live within walking distance of the office.

The participants of the study were home care services in two municipalities, A and B, in northern Norway. Municipality A has a total land area of 405.58 km^2^ (156.60 square miles), and a population of 10 800. Municipality B has a total land area of 698.22 km^2^ (269.58 square miles), and a population of 10 000. Of the inhabitants, 16% in municipality A and 13% in municipality B are 67 years and older.Home care services in municipality A are organized into five sectors and in municipality B into four. Municipality A has located its five home care services offices in five smaller municipal centers, while municipality B has located them all in one place, the community center (see Figure 1).

We collected data from those offices from where the employees mostly visited home care recipients, using a car. In municipality A, this was four offices, numbered 1, 2, 3, and 4, and in municipality B, it was three, numbered 1, 2, and 3. In the week of the study, 276 home care recipients in municipality A and 181 in municipality B had one or more visits. Table 1 shows the number of home care recipients visited in each sector.

**Table 1:** **Number of home care recipients visited during the week of the study**

	All	Office 1	Office 2	Office 3	Office 4
Municipality A	276	81	77	45	73
Municipality B	181	82	49	50	

### Data collected for this study

We collected 420 day schedules from home care services offices in the two municipalities, covering a period of 1 week in December 2013. In addition, we recorded 471 decisions describing how much time was allocated to each individual home care recipient each week.

In municipality A, schedules only give the duration of the visits where those are fixed for some reason, unlike municipality B, where the duration is given for all visits. We had to go through all the vehicle routes with a divisional nurse for every sector in municipality A to find the time scheduled for each visit.

To estimate how far each employee drove and the driving time required on day and evening shifts, all day schedules and driving routes for Monday to Sunday were registered in Excel for analysis using GIS. Visits were recorded in the order in which they were set up by the home care services office. For each visit, the nurse or health worker must spend time to park the car, find the necessary equipment, and time of entering the house. After the visit, he spends time on going out again and back to the car. The worker will spend some time on this depending on how far from the house he has to park the car. After discussion with divisional nurses we added five minutes transfer time, for the time spent getting out of the car and into the house, and then out of the house and into the car. We were therefore able to estimate the total driving time. The visit time for each user was also recorded, as well as the working time including shift reports (handover time) and breaks for each employee. Shift reports lasted 30 minutes in the morning in both municipalities, but differed in the afternoon. Municipality B had a 10- to 15-minute report on the afternoon shift, while reporting time in municipality A varied between 10 and 30 minutes.

### Analysis of the data

Data were analyzed using ArcGIS version 10.1, Network Analyst, a GIS. GIS are computer-based systems for storing, managing, analyzing, and presenting geographic data [33]. They are “enabling” technology tools, used for working with data with a spatial component. For home care services, the spatial components were the offices and patients’ addresses.

**Figure 2:**
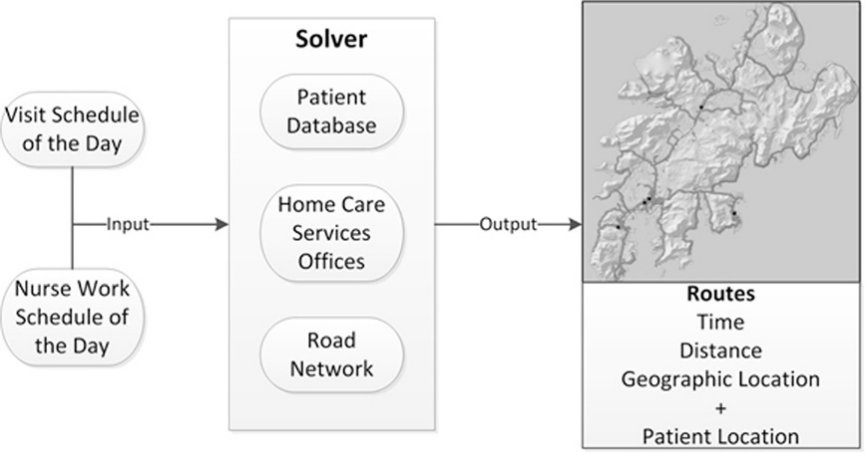
**The ‘Solver’** [34] **.**

Using ArcGIS version 10.1, Network Analyst, a basemap with a street network containing speed limits and distances for all streets was created for both municipalities. The speed limits and distances were necessary to calculate the routes as realistically as possible. Each patient’s address and the home care services offices were included on the basemap. The addresses and any other patient-identifying information were removed from the data set before further analysis to ensure patient confidentiality. Based on the location of the home care services office(s), a service area analysis was carried out, to show the area an employee can cover from his/her office, in terms of driving time.For the routing analysis, a model was created (Figure 2) for calculating each route, called the “solver”. All driving routes were recorded in the solver. Based on this information, the solver took into consideration the location of patients and home care services office, and the road network, and calculated the best route. The final routes were displayed on a map, and a table showed the time required for each route and its distance.

The required routes for each day can be displayed graphically on the basemap once calculated. Using ArcGIS Server terms, the application required mapping, feature, geoprocessing, and geocoding services. These services were developed from map documents made in ArcMap 10.1. The mapping service produced the basemap for the project. It contained layers with geographic reference information, useful for viewer orientation. Those layers that had operational functions, such as searching and exploring attributes, could be published as a feature service. A geocode service was added to make it easier to explore the map based on address location.

### Methodological considerations

In order to obtain reliable findings, it was important that all map data was correct, including addresses and speed limits on individual roads. Local maps were not updated with new addresses, so we needed some verification. Where addresses were not clear, we checked with the employees responsible for route planning. To check the speed limits, where necessary we drove the routes.

### Ethical considerations

The research project was supervised by the Regional Committee of Ethics (Northern Norway) in 2011 and the study was done by following the Helsinki Declarations ethical guidelines. The registration number from the Regional Committee of Ethics is 2011/2457-6 (Document-id is number 181546). Since the data material is indirectly person-identifiable, it has been anonymized. Notification of the project was sent to the Norwegian Social Science Data Services, and the study was advised in 2011 with the reference number 28643. Verbal information and written approval were given as described in the Helsinki Declaration.

## Results

In total, in the week examined, there were 276 home care recipients in municipality A and 181 in municipality B. Staff carried out 2 677 visits in municipality A, and 1 466 in municipality B. The employees drove 268 scheduled tours in municipality A and 152 tours in municipality B (Table 2).

**Table 2:** **Overview of the number of home care recipients, visits, and driving routes for each office**

HCS office	Municipality A			Municipality B		
	Recipients	Visits	Routes	Recipients	Visits	Routes
**Office 1**	81	528	61	82	519	57
**Office 2**	77	888	82	49	493	47
**Office 3**	45	691	64	50	454	48
**Office 4**	73	560	61			
**Total**	**276**	**2667**	**268**	**181**	**1466**	**152**

On average, every employee visited 10 home care recipients during their shift, although the actual planned number of visits ranged from one to 23 in a single shift. The employees had most planned visits on the afternoon and evening shift.

### Location of home care recipients – travel time from home care services offices

From the GIS analysis, we obtained a map showing the location of all home care recipients and home care services offices. Figure 1 shows the distance which can be travelled from each office in 5, 10, 15, 20, and 30 minutes.Figure 1 does not make clear that B has a bigger area than A, since it only shows the road network, and not the actual landscape. However, the figure does show the concentration of home care recipients, and how far they are in driving time from a home care services office. Most of the home care recipients live within 10 minutes’ drive of the local office. The GIS maps show why the municipalities organize home care services differently. Municipality A was once four municipalities, which merged in 1963. It therefore has four obvious smaller centers. Municipality B has one obvious location for the office at the center.

### Driving routes

**Figure 3:**
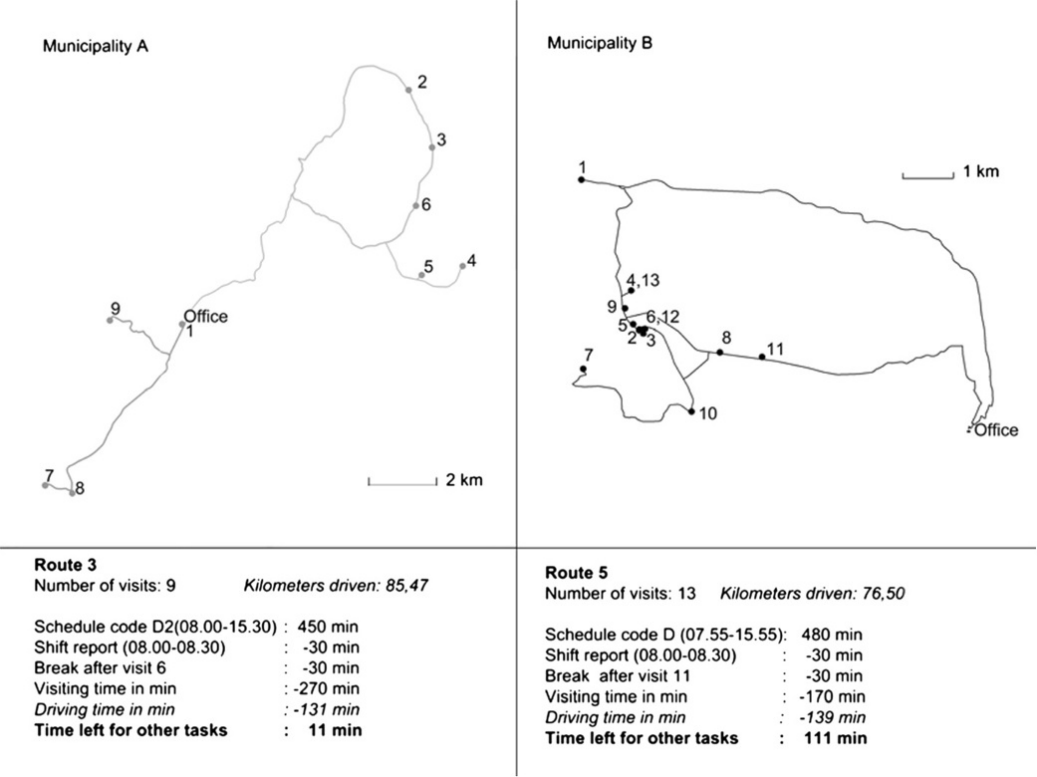
**Example driving route from each municipality, from GIS.** The panels show the number of visits, kilometers driven and time consumption.

The number of planned visits on each route varied from day to day through the week on the day shift, although the number of visits on the evening shift was about the same each day.Use of GIS for driving route analysis gives a picture of geographic accessibility to the home care recipients. Figure 3 shows a map of two of the planned driving routes, one from each municipality on a weekday. The maps show the distance from the home care services office to the individual home care recipients. The planned driving route in Municipality A showed that there were only 11 minutes left to do other tasks like writing legally-required documentation. The work on this route was to help home care recipients with morning care, showering, wound care, drug administration, and putting on compression stockings. In municipality B, the employee had 110 minutes to do other tasks. The work on this route was to help home care recipients with drug administration, put on compression stockings, provide morning care, and wound care. Two home care recipients got two visits because they needed help with breakfast and dinner. The maps also showed that the driving routes were planned in a way that the employees had to drive the same route in different directions several times.

### Kilometers driven

The planned driving routes in Municipality A showed that the employees had to drive a total distance of 7 933 kilometers, with each one averaging 29.60 km. In municipality B, the planned driving routes had a total distance of 5 959 km and an average of 39.20 km.The map in Figure 2 shows that in municipality A, there is a large difference in the size of the land area covered by each office, which is reflected in how many kilometers employees have to drive on average to visit home care recipients. Employees at Office 1 drove the largest number of km during the week, 3 450 km. The staff at Offices 2, 3 and 4 drove 2 040 km, 1 209 km, and 1 234 km, respectively. In municipality B, employees drove 2 653 km, 1 559 km, and 1 747 km from Offices 1, 2, and 3.

In municipality A, there were two sectors, 2 and 3, where most driving routes were less than 10 km. In sector 1, most driving routes were 10 km or more and in sector 4, the majority of employees drove between 10 and 20 km during the shift. Only sectors 1 and 2 had any driving routes between 100 and 120 km.

Municipality B had a different driving pattern from municipality A (Table 3). All sectors had routes that were less than 10 km, but in sector 1, the majority of the driving routes were between 40 and 70 km. For sectors 2 and 3, most routes were respectively 30–40 km and 20–30 km.

**Table 3:** **Ratio of short and long driving routes in Municipalities A and B**

	Municipality A				Municipality B		
	Sector 1	Sector 2	Sector 3	Sector 4	Sector 1	Sector 2	Sector 3
Kilometers	Number of routes n%	Number of routes n%	Number of routes n%	Number of routes n%	Number of routes n%	Number of routes n%	Number of routes n%
**0-10 km**		46 (56%)	29 (45%)	12 (20%)	8 (14%)	11 (23%)	8 (17%)
**10-20 km**	3 (5%)	9 (11%)	7 (11%)	22 (36%)	4 (7%)	6 (13%)	4 (8%)
**20-30 km**	10 (16%)	1 (1%)	8 (13%)	15 (25%)	2 (4%)	6 (13%)	13 (27%)
**30-40 km**	4 (7%)		8 (13%)	10 (16%)	6 (11%)	13 28%)	5 (10%)
**40-50 km**	9 (15%)	2 (2%)	11 (17%)	2 (3%)	9 (16%)	2 (4%)	
**50-60 km**	14 (23%)	8 (10%)	1 (2%)		8 (14%)	1 (2%)	2 (4%)
**60-70 km**	3 (5%)	7 (9%)			9 (16%)	1 (2%)	2 (4%)
**70-80 km**	8 (13%)	1 (1%)			6 (11%)	6 (13%)	1 (2%)
**80-90 km**	6 (10%)				3 (5%)		2 (4%)
**90-100 km**		7 (9%)			2 (4%)		1 (2%)
**100-110 km**	2 (3%)	1 (1%)					
**110-120 km**	2 (3%)					1 (2%)	
**120-130 km**							1 (2%)
**Total**	**61 (100%)**	**82 (100%)**	**64 (100%)**	**61 (100%)**	**57 (100%)**	**47 (100%)**	**48 (100%)**

### Spatial analysis

**Figure 4:**
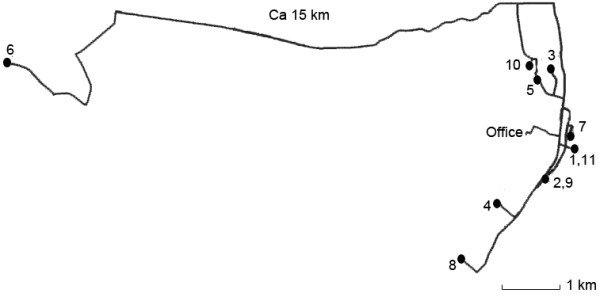
**Example of driving route involving home care recipient living some distance apart.**

Spatial analysis of the planned driving routes related to care given showed that 5% of the driving routes in municipality B were set up in such a way that the employee visited one or two home care recipients living some distance apart. Figure 4 shows such a route, where the employee drove 15 km each way to visit a home care recipients for just 15 minutes. All other home care recipients were living in the same area. The weekly plan showed that there were two other employees who drove the same way, one of whom could have made this visit instead. In municipality A, only 2% of the driving routes were set up in this manner.

### Allocation of working time

Using GIS, we calculated the expected driving time. Visiting times were allocated and the remaining working time was for other necessary tasks, such as legally-required documentation, home visits to people who have applied for home care services, and a statutory break. Table 4 shows how much time the employees spent on driving, visits, completing patient documentation and other tasks during the week. It shows that driving time in both municipalities was approximately 20% of the total shift time. In municipality A, driving time varied between 18% and 25% of total shift time, and in municipality B, it was between 21% and 22%. Visiting time varied between 44% and 69% in municipality A, and between 40% and 56% in municipality B. Other tasks, including the legally-required documentation of the care given, varied between 10% and 32% in municipality A and 24% and 38% in municipality B.

**Table 4:** **Allocation of working time during the week (n is the sum of the normal working hours the employees were scheduled to work that week in each office)**

	Municipality A				Municipality B		
Office	Office 1	Office 2	Office 3	Office 4	Office 1	Office 2	Office 3
Working hours	Hours (n = 401.5)	Hours (n = 517.25)	Hours (n = 414)	Hours (n = 402.5)	Hours (n = 397.5)	Hours (n = 322)	Hours (n = 319.5)
**Driving time**	100.83 (25%)	109.38 (21%)	74.27 (18%)	74.00 (18%)	88.17 (22%)	70.93 (22%)	66.58 (21%)
**Visiting time**	177.67 (44%)	356.75 (69%)	212.00 (51%)	199.67 (50%)	159.08 (40%)	170.38 (53%)	177.97 (56%)
**Time to other tasks**	123.00 (31%)	51.12 (10%)	127.73 (31%)	128.83 (32%)	150.25 (38%)	80.68 (25%)	74.95 (23%)
**Total**	**401.50 (100%)**	**517.25 (100%)**	**414.00 (100%)**	**402.50 (100%)**	**397.50 (100%)**	**322.00 (100%)**	**319.50 (100%)**

The employees use mobile devices such as tablets or mobile phones to retrieve information about home care recipients. With mobile devices, employees can complete the legally-required documentation after each visit. The manager of home care services in both municipalities sees it as an advantage that all reports are written immediately after each visit, although this time is not set aside in the weekly plan. Writing legally-required documentation takes some time depending on what care has been provided and how familiar the employee is with the mobile device. In this study, we examined whether managers need to take into account time spent on documentation. We estimated that this took 5 minutes per visit, on average, which was confirmed by the offices. When we added 5 minutes for documentation onto each visit, the total time employees spent on visiting home care recipients increased by 10% at Office 1, 14% at Offices 2 and 3, and 12% at Office 4 in municipality A, and by 11% at Office 1, 13% at Office 2, and 12% at Office 3 in municipality B (Table 5). Time to do other tasks was reduced accordingly.

**Table 5:** **Allocation of working time during the week, with documentation time included in visiting time (n is the sum of the normal working hours the employees were scheduled to work that week in each office)**

	Municipality A				Municipality B		
Office	Office 1	Office 2	Office 3	Office 4	Office 1	Office 2	Office 3
Scheduled working hours	Hours (n = 401.5)	Hours (n = 517.25)	Hours (n = 414)	Hours (n = 402.5)	Hours (n = 397.5)	Hours (n = 322)	Hours (n = 319.5)
**Driving time**	100.83 (25%)	109.38 (21%)	74.27 (18%)	74.00 (18%)	88.17 (22%)	70.93 (22%)	66.58 (21%)
**Visiting time including documentation**	218.58 (54%)	430.75 (83%)	267.83 (65%)	246.33 ( 61%)	202.33 (51%)	210.63 (65%)	215.88 (68%)
**Time to other tasks**	82.08 (20%)		71.90 (17%)	82.17 (20%)	107.00 (27%)	40.43 (13%)	37.03 (12%)
**Total**	**401.49 (100%)**	**540.13 (104%)**	**414.00 (100%)**	**402.50 (100%)**	**397.50 (100%)**	**321.99 (100%)**	**319.49 (100%)**

In municipality A, at office 2, if the planned allocation of work to the employees had incorporated the extra 5 minutes spent writing the statutory documentation after each visit, then the total time spent at work would have exceeded the original plan (Table 5).

### Overtime

By examining each driving route for the week, including driving time, it was clear that some routes were planned with insufficient time to do the scheduled tasks (see Tables 6 and 7).

**Table 6:** **Percentage of routes where the work can be done within normal working hours**

Municipality	Municipality A	Municipality B
Routes	Routes (n = 268)	Routes (n = 152)
**Normal working hours**	209 (78%)	131 (86%)
**Overtime**	59 (22%)	21 (14%)
**Total**	**268 (100%)**	**152 (100%)**

**Table 7:** **Percentage of routes where the work can be done within normal working hours when writing the legally required documentation after each visit**

Municipality	Municipality A	Municipality B
Routes	Routes (n = 268)	Routes (n = 152)
**Normal working hours**	169 (63%)	103 (68%)
**Overtime**	99 (37%)	49 (32%)
**Total**	**268 (100%)**	**152 (100%)**

In municipality A, 22% of the driving routes were planned with insufficient time to do the scheduled tasks. Taking into account the time required to write up the statutory documentation of care given, the number of routes with insufficient time allocated increased to 37% (Tables 6 and 7).

For municipality B, there were fewer routes that were planned with insufficient time to do the scheduled tasks. Without including time for statutory documentation, only 14% were planned with insufficient time to do the scheduled tasks. Including the statutory documentation time; however, 32% of routes were planned with insufficient time to do the scheduled tasks.

## Discussion

Using the maps, it is easier to understand why it is important to take into account the driving time. Tables 4 and 5 show there is a range of variability in the driving time, in the visiting time, and in the time taken for other tasks such as legally-required documentation between municipality A and B. There are several reasons for these variations. The difference related to driving time between sectors in municipality A is because of the different distances and the numbers of visits. The variations in visiting time are caused by how many home care recipients each sector was assigned. It also depends on whether the office managers can change the written assignment if the home care recipients require fewer or more hours each week. The home care services in both municipalities have a fixed number of personnel. The assignments change frequently for various reasons. Home care recipients require more help when they get sick and none at all if they temporarily move into a hospital or a nursing home. If a home care recipient assigned 20 hours a week in care moves to a nursing home or dies, then the sector gains that 20 hours for other tasks. Municipality B has a greater opportunity to deal with variations in visiting time between sectors because the sector offices are in the same place. The sector managers have a policy of helping each other with the daily program. In municipality A, it is harder to deal with visiting time variations. The sectors do not overlap, and the sector managers have only one weekly meeting with the service manager. If an employee in Office 3 has only a few visits and Office 1 needs help, the distance between the offices is 18 km, and the home care recipient may live 15 km from the office.

Maps that show where home care recipients live give a picture of the accessibility of delivering home care services. Our study found that geographic factors, particularly driving time, including transfer time, affect how much time employees spend with home care recipients. If we look at how the driving time, visiting time, and time for other tasks are planned related to the total working time for each office, it appears that there is sufficient time to do the daily work (Table 4). However, analyzing each driving route using GIS gives a different picture: then, both municipalities had planned routes with too many visits.

By taking into account the transfer time as well as time in the car, we got a more accurate picture of the total time taken to get between home care recipients’ visits. Employees did not consider transfer time to be important, but it can add up to quite a large proportion of the working day. If the employee visits 10 home care recipients, the transfer time could add up to 50 minutes, if it is 5 minutes per visit. In the winter with a lot of snow, the transfer time may be longer. If the scheduler does not take transfer time into account, he or she may think the employee has more time available than is actually the case.

If there are no unexpected events during visits, it seems likely that planned working hours are sufficient for all the visits. However, the afternoon/evening shift workers have a particularly tight schedule. When something unexpected happens, for example, a home care recipient has fallen and must be hospitalized, the employee seldom submits written admission notes to the hospital in accordance with best practice to ensure continuity of care [35]. Olsen et al. [35] found that admission notes were present in only 1% of patient transfers from home care to hospital. This may indicate that other municipalities also have tight schedules. Other studies have shown that employees prioritize meeting recipients’ needs rather than compliance with documentation requirements [11, 16].

As mentioned in the introduction, many employees of home care services experienced increased time pressure and lack of time as a dominant and restricting factor in providing services [11–16]. If driving time is not taken into account, it is easy to plan too many visits for one route. There is also a problem that the assignments of hours to home care recipients are not always updated [36, 37]. If the weekly plans do not show the number of hours the home care recipients really need, the office manager needs to tell the staff about changes.

The use of smartphones and tablet PCs, the home care services manager in both municipalities expects that employees will write their statutory report of the care given immediately after the visit, and not when they return to the office. When the employee writes their report immediately, taking on average 5 minutes, the timing of the remaining visits will be delayed by 5 minutes for each visit. By introducing this way of working, it is possible that the order of visits will need changing, especially if recipients need to be visited at a particular time.

There is an expectation that the use of smartphones and tablet PCs will release more time for home care recipients. A study from Copenhagen showed that the use of netbooks to document the care given after each visit reduced the time spent on report-writing by an average of 15 minutes per day per employee [38]. Use of netbooks proved to be good for visits of longer duration. For short visits, however, it took too long to log in and out of the netbook, while the focus was efficiency. Mobile ICT devices are here to stay, but there has, as yet, been no research showing how ICT changes the working day in home care services.

Overtime on some schedules and driving routes is a problem in both municipalities, primarily for evening routes and short shifts. During this particular week, Municipality A had a real problem with overtime. Analyzing the maps, it was largely Office 2 that was the problem, because several employees were off sick with flu, and it was difficult to find temporary substitutes.

If the time taken for statutory documentation of care given is taken into account, overtime seems to be a greater challenge to both municipalities. Employees on these routes were not able to spend the designated time with the home care recipients and stay within given working hours. On the day shift, it seems to be possible to coordinate driving routes so that all work is done during working hours, but it is harder on the evening shift. This is less of an issue in municipality B than municipality A, because all the home care services offices in B have home care recipients in the municipality center. The offices cooperate about visiting home care recipients, and the managers talk over the problem, and change the schedules. This is more difficult in municipality A because of the distance between offices and because the managers only meet each other once a week, along with the service manager.

The consequence of setting up day schedules and driving routes with too many visits in relation to working hours is that home care recipients do not get the service they require, and have been allocated, and the employees do not have time to do all the necessary work. If something unexpected happens on the shift, it is not certain that all visits will be carried out. To have a strict schedule creates frustration and stress for employees [11, 39, 40]. With schedules that are over-strict in relation to working hours, it will be difficult for the employees to follow up the requirements in *The Coordination Reform: Proper treatment – at the right place and right time* [3] and *Regulation of Quality of Care in Health and Social Services Regulated in the Municipal Health Care Act* and the *Social Health Care Act* [41].

As spatial analysis shows, some driving routes need to be better organized to reduce unnecessary driving. Fuel in Norway costs approximately NOK 16 per liter, NOK 60 per US gallon (about $10 or £6), so good organization of routes could reduce ongoing vehicle costs, and therefore the cost of the service overall.

## Conclusions

Using GIS to analyze driving routes in home care services, our study shows that driving time and time required to write up statutory documentation are underestimated in planning weekly day schedules. In addition, the maps shows that municipal geography determines where home care services’ sector offices are located. About a third of day schedules are set up with too many visits, so that employees do not have time to complete all that is required. More research is needed to gain knowledge of how time is allocated to home care recipients, particularly in countries where home care is a free service, to get more visit time and reduce driving time.

### Implications for clinical practice

There are many people who will need home care services in the future, and a fair distribution of services must be managed. One way to do that is to improve knowledge of time requirements, and to develop planning tool with a convenient user interface that is integrated into the patient documentation system. Our study suggests that GIS could help as a planning tool in these services and as a result provide control over the time spent related to visits to home care recipients.
